# Structure Driven Tuning of the Catalytic Performance of PtCe-Modified Zeolite ZSM-5 in the CO Oxidation

**DOI:** 10.3390/molecules31010156

**Published:** 2026-01-01

**Authors:** Marina Shilina, Irina Krotova, Konstantin Maslakov, Stanislava Petrova, Olga Udalova, Tatiana Rostovshchikova

**Affiliations:** 1 Chemistry Department, Lomonosov Moscow State University, 119991 Moscow, Russiartn@kinet.chem.msu.ru (T.R.); 2 Semenov Federal Research Center for Chemical Physics, RAS, 119991 Moscow, Russia

**Keywords:** zeolite ZSM-5, platinum, cerium, catalysis, carbon monoxide, oxidation

## Abstract

The catalytic oxidation of CO is of great technological importance for the treatment of vehicle and industrial exhaust gases. PtCe-catalysts of low-temperature CO oxidation were prepared by the impregnation of ZSM-5 zeolite (Z) with aqueous solutions of H_2_PtCl_6_ and Ce(NO_3_)_3_, varying the order of metal deposition and thermal treatment conditions. The relationships between structure transformations and catalyst performance were established based on the SEM, TEM, EDX, DRIFT, and X-ray photoelectron spectroscopies data. For the Ce/Pt/Z sample, in which cerium was deposited after platinum, the 100% CO conversion temperature was only 120 °C. The inverse deposition sequence of metals (Pt/Ce/Z catalyst) resulted in CO oxidation at a higher temperature that can be decreased to 110 °C by redox treatment. The prepared catalysts were also active in the CO oxidation in excess hydrogen (PROX) but were not selective enough. However, the activity of PtCe-modified ZSM-5 enhanced greatly in the repeated cycles of CO oxidation (TOX) after testing in PROX. It is suggested that enhancing the interaction between Pt and Ce is a key factor in tuning the catalyst performance. The 0.2 wt.% Pt catalysts showed the best performance and provided complete CO conversion at 95 °C, which is a pronounced result for low-loaded Pt catalysts.

## 1. Introduction

The catalytic oxidation of CO is of great technological importance for the treatment of vehicle and industrial exhaust gases and for the removal of CO impurity from hydrogen supplied to fuel cells [1,2,3]. CO oxidation has been of fundamental interest as a model reaction to analyze the relationship between structure and catalytic performance, which is important for the rational design of efficient catalysts for other oxidation reactions [4,5,6]. Supported noble metal catalysts, especially platinum, are widely used in the environmentally important combustion of CO, hydrocarbons, and other volatile organic compounds (VOCs) [7,8,9,10,11,12]. Studies in this field are mainly aimed at improving low-temperature activity and increasing selectivity and stability of the catalysts [13,14,15,16]. A complete CO conversion is typically achieved at temperatures below 150 °C for catalysts with Pt loadings above 1% [17,18,19,20,21], and only a limited number of works report improved catalytic properties for low-loaded Pt-based systems [22,23,24]. CO oxidation over monometallic Pt catalysts proceeds only at elevated temperatures due to a strong CO adsorption on highly dispersed Pt particles [25]. The presence of partially oxidized platinum in the active sites results in reducing unwanted CO adsorption and shifting the oxidation process to the low temperature region [26,27,28]. The use of suitable supports or promoters makes it possible to tune the dispersion and electronic state of the metal on the catalyst surface and thereby improve catalytic performance [29,30,31,32,33]. Due to their unique physicochemical properties, zeolites are excellent supports for highly dispersed metal species with improved catalytic properties [16,34,35,36]. Pt interaction with acidic centers of zeolites leads to the formation and stabilization of catalytically active PtO_x_ species, which ensure high efficiency of the zeolite-supported catalysts [37,38,39]. Another well-known way to tune the electronic state of platinum group metals is to use easily reducible supports, among which ceria is especially important [40,41,42]. Ceria-supported Pt catalysts are applied as exhaust converters and fuel cell catalysts [43,44]. The metal-oxide interaction plays a key role in ensuring high-performance of these systems [33,40,41,42,44,45]. The efficiency of the metal-oxide interaction strongly depends on the chemical state of the metal in the catalyst, which is determined by many factors such as the synthesis method, metal loading, oxide support nature, treatment in a reducing or oxidizing atmosphere, and reaction conditions [20,24,46,47]. Due to the dynamic transformations of various metal forms ranging from metallic or oxidized single atoms, clusters, and nanoparticles under process conditions, their role in catalysis is still debated. However, it has been clearly established that the interfacial Pt−O−Ce bonds anchoring the Pt to the CeO_2_ surface play a key role in the oxygen transfer process [8,28,33,48]. Redox processes at the metal-oxide interface provide the charged species that change the mechanism of CO oxidation from the Langmuir–Hinshelwood to the Mars-van Krevelen process with the participation of oxygen vacancies of CeO_2_ [32,49,50,51]. Moreover, steam treatment or H_2_O admixtures in the reaction mixture has a promoting effect on the PtCe catalysts via changes in their surface structure that provide a new way for CO oxidation with the participation of surface OH groups [52,53,54].

Compared to a bulk support, the metal-oxide interactions are even more significant when Pt species are anchored to CeO_2_ nanoparticles [45,54]. The zeolite-supported PtCe catalysts exhibited improved performance in several industry-important processes [55,56]. Recently, ZSM-5 zeolite-supported low-loaded PtCo catalysts demonstrated an improved performance in CO-PROX due to a synergistic effect of Pt and Co species closely located in zeolite channels [38,39,57].

The aim of this work is to optimize the synthesis of PtCe-modified zeolites comparing their catalytic activity in the CO-TOX and PROX reactions. The properties of the synthesized PtCe-modified zeolites were also tuned via different redox treatments. The relationship between structure transformations and catalyst performance was established based on the data of SEM, TEM, EDX, DRIFT-, and X-ray photoelectron spectroscopies. The activity of PtCe-modified ZSM-5 in the CO oxidation (TOX) enhanced greatly after testing in the H_2_ excess (PROX). The 0.2 wt% platinum catalyst showed the best performance and provided complete CO conversion at 95 °C, which is a pronounced result for a low-loaded catalyst.

## 2. Results

**Figure 1:**
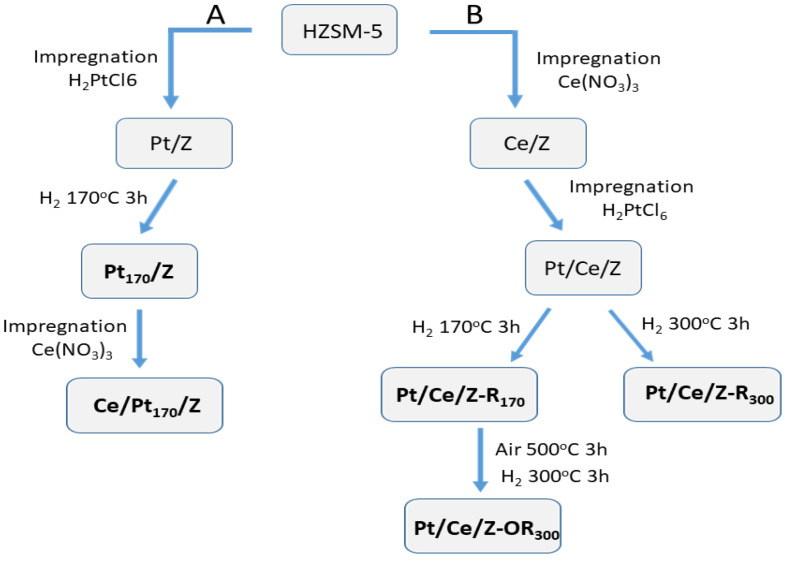
Scheme of catalyst synthesis and post-synthetic treatments: (path **A**), Pt was deposited first, (path **B**), cerium was deposited first.

Bimetallic Pt-Ce-modified zeolites (Z) were synthesized by sequential impregnation of zeolite ZSM-5 (SiO_2_/Al_2_O_3_ = 55) with water solutions of H_2_PtCl_6_ and Ce(NO_3_)_3_ using different sequences of impregnation and post-synthetic redox treatments as described in Section 4.1. The scheme of catalyst synthesis and post-synthetic activation in accordance with A and B protocols is shown in Figure 1. Bimetallic Ce/Pt_170_/Z catalyst was prepared from Pt_170_/Z preliminary reduced with H_2_ at 170 °C. Pt/Ce/Z-R_170_ and Pt/Ce/Z-R_300_ zeolites with the reverse order of zeolite impregnation with metal salts were reduced at 170 and 300 °C, while Pt/Ce/Z-R_170_ was additionally subjected to oxidation–reduction treatment, firstly in a stream of air at 500 °C and then in a stream of hydrogen at 300 °C.

### 2.1. DRIFT Spectroscopy of the Adsorbed CO

**Figure 2:**
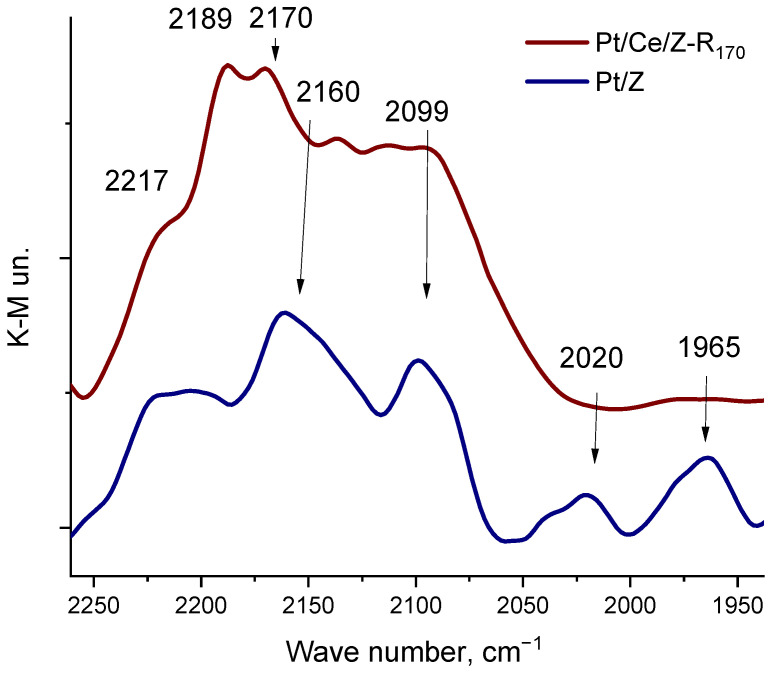
DRIFT spectra of carbon monoxide adsorbed at RT on monometallic Pt_170_/Z and bimetallic Pt/Ce/Z-R_170_ catalysts at equilibrium pressures of CO 2.5 kPa.

The electronic and coordination states of platinum and cerium on the zeolite surface were studied by the DRIFT spectroscopy of adsorbed carbon monoxide that relies on the high sensitivity of the CO stretching frequency to the structure of binding sites as described in [38,58]. In the spectrum of CO adsorbed on monometallic Pt_170_/Z (Figure 2), along with absorption bands at 2217 and 2190 cm^−1^, corresponding to the Lewis acid centers of zeolite [59,60], new bands are observed in the region of 2160–1900 cm^−1^. These bands are absent in the spectra of unmodified zeolite at these CO pressures. A relatively broad band in the region of 2160 cm^−1^ is due to CO adsorbed in the form of mono- and polycarbonyls on oxidized platinum Pt^n+^ species, where *n* ≥ 1 [59,61,62]. Absorption bands in the region of 2100 cm^−1^ in the Pt/Z spectrum may correspond to the linear adsorption of CO on isolated platinum atoms or Pt^δ+^ species_,_ and broad absorption bands at 2027 and 1965 cm^−1^ may be associated with the linear and bridging adsorption of CO on platinum clusters or nanoparticles, respectively [63,64]. Thus, the monometallic Pt_170_/Z catalyst contains oxidized and partially reduced isolated platinum atoms and Pt metal clusters or nanoparticles.

Modification of the zeolite surface with cerium increases the platinum resistance to aggregation. Figure 2 shows the spectrum of CO adsorbed on the Pt/Ce/Z-R_170_ sample under the same conditions as for the monometallic one. An intense broad band with a maximums at 2189 cm^−1^ corresponds to the CO adsorption on cerium cations located in the ion-exchange positions of the zeolite, mainly in the form of Ce^3+^ or Ce^4+^ hydroxocations or a small fraction of oxocations [65], a band at about 2170 cm^−1^ may be attributed to CO adsorbed on cerium oxide CeO_2_ [59]. In this case, there are no CO bands in the IR spectrum in the low-frequency region (below 2100 cm^−1^) that can be related to the adsorption of CO on platinum clusters or nanoparticles. The high resistance of platinum to aggregation during reduction treatment in the presence of cerium was also confirmed by the microscopy analysis.

### 2.2. XRD and Microscopy Analysis

XRD data for the initial zeolite (Z) and an example of PtCe-modified zeolite are presented in Figure S1. The XRD pattern of Z is typical for ZSM-5 zeolite (JCPDS: 00–044-0003), and no reflections referring to other phases can be found. All the characteristic zeolite peaks are also visible in the XRD pattern of Pt/Ce/Z indicating that the crystal structure of the zeolite remains unchanged upon the modification. Only weak reflections corresponding to the cubic CeO_2_ phase appear in the XRD pattern of Pt/Ce/Z (JCPDS: 43–1002). Reflections corresponding to platinum species are absent due to the high dispersion and low content of these species.

**Figure 3:**
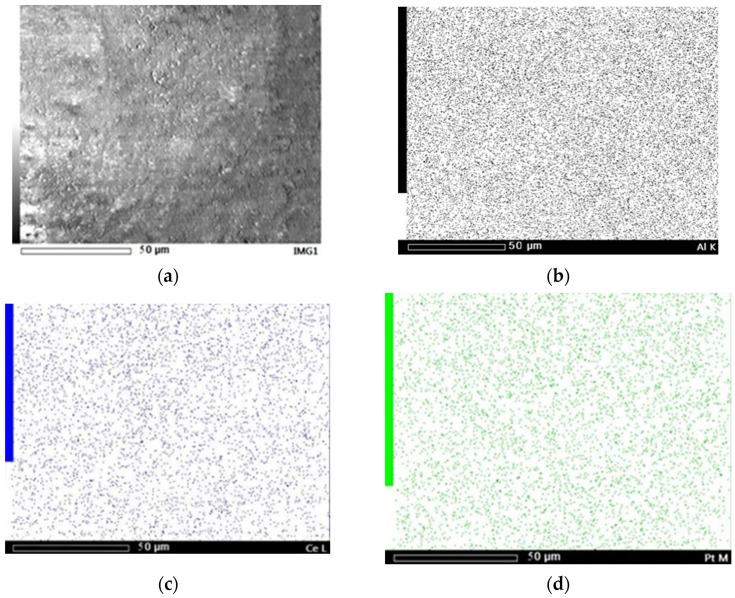
SEM image (**a**) and Al (**b**), Ce (**c**), and Pt (**d**) SEM-EDX mappings of Ce/Pt_170_/Z.

Typical SEM image of the Ce/Pt_170_/Z surface and the corresponding SEM-EDX element mappings are shown in Figure 3. As one can see, Pt and Ce demonstrate the same uniform surface distributions; thus, the sequential introduction of components ensured their proximity on the catalyst surface.

EDX surface compositions of mono- and bimetallic samples prepared by different methods (Figure 1A,B) are summarized in Table 1. When cerium was introduced after platinum, the Pt content on the Ce/Pt_170_/Z surface is almost 2 times lower than in the monometallic Pt_170_/Z catalysts and in the Pt/Ce/Z-R_170_ catalyst synthesized using the reverse order of metal introduction. The cerium content is almost identical in both cases.

**Figure 4:**
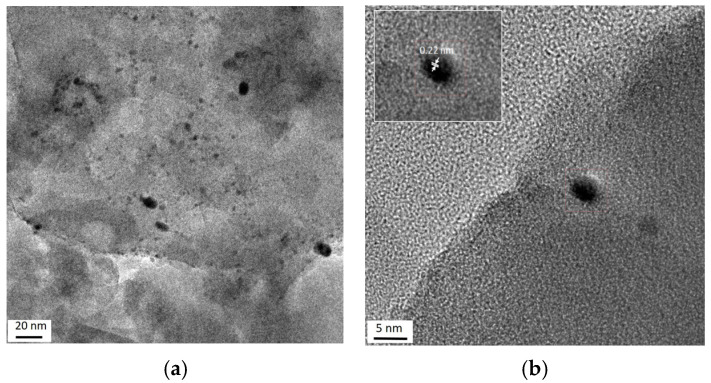

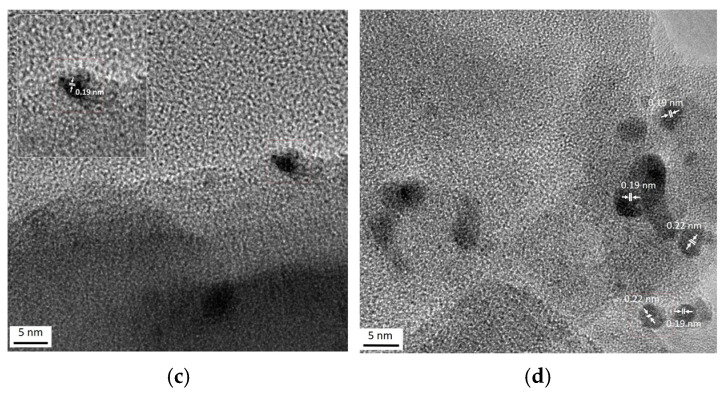
TEM (**a**) and HR-TEM (**b**–**d**) images of monometallic Pt_170_/Z sample.

Figure 4 shows typical TEM images of the monometallic Pt_170_/Z sample. The interplanar spacings in platinum-containing particles (0.14, 0.19 and 0.22 nm) are close to those of (200) and (111) planes of metallic Pt (JCPDS 70-2057).

**Figure 5:**
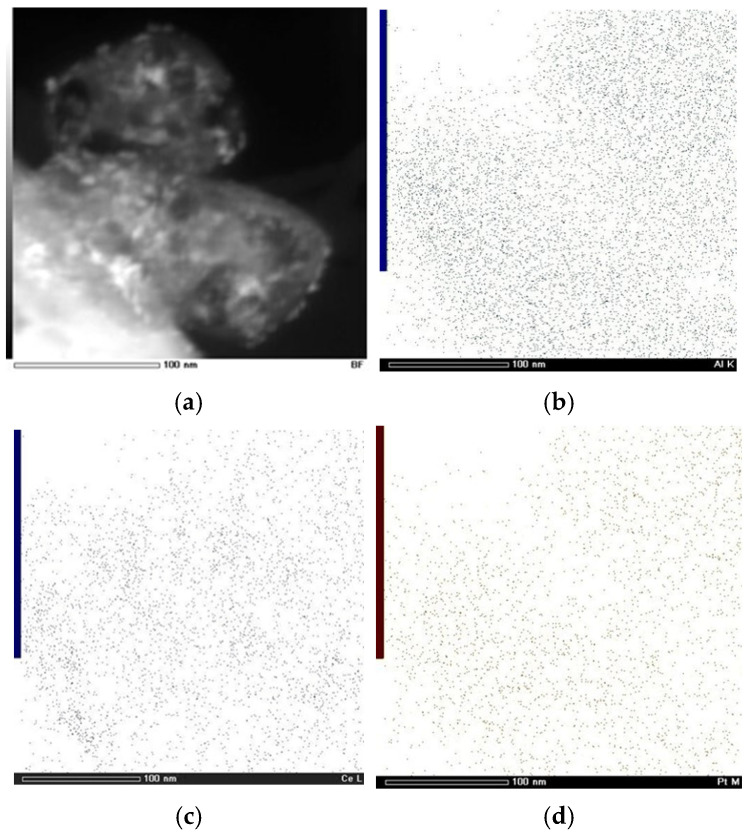
TEM image (**a**); TEM-EDX mapping of Al (**b**), Ce (**c**), and Pt (**d**) for the Pt/Ce/Z-R_300_ sample.

**Figure 6:**
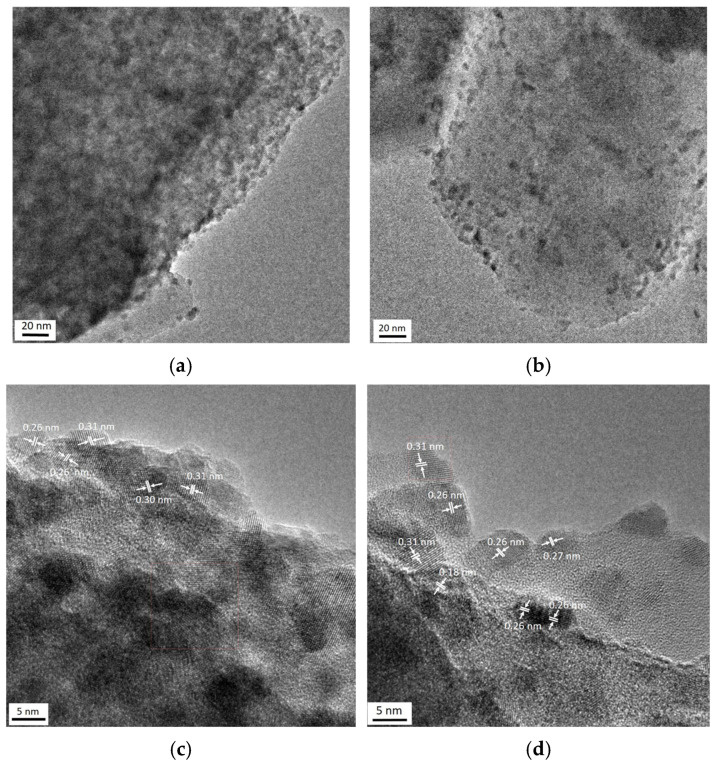
TEM (**a**,**b**) and HR-TEM (**c**,**d**) images of Pt/Ce/Z-R_300_.

The TEM results for the bimetallic catalysts are shown in Figure 5 and Figure 6. The TEM-EDX element mappings also confirm the same along with uniform distributions of cerium and platinum on the zeolite surface (Figure 5). Highly dispersed species observed in the TEM images (Figure 6a–d) may be associated with both Pt- and Ce-containing particles. Some regions of ordered atoms that are clearly visible in images (Figure 6c,d) can be related to the (111) and (200) planes of CeO_2_ (JCPDS: 43–1002). Moreover, there are other regions with interplanar distances of 0.26 and 0.18 nm that are close to the (200) and (220) planes of PtO (JCPDS 47-1171). This fact distinguishes the bimetallic samples from the monometallic ones, where the interplanar distances typical for metallic platinum were detected (Figure 4b–d). The absence of metallic platinum nanoparticles in the bimetallic samples even after reduction at 300 °C agrees with the IR spectroscopy data.

### 2.3. XPS Data

**Figure 7:**
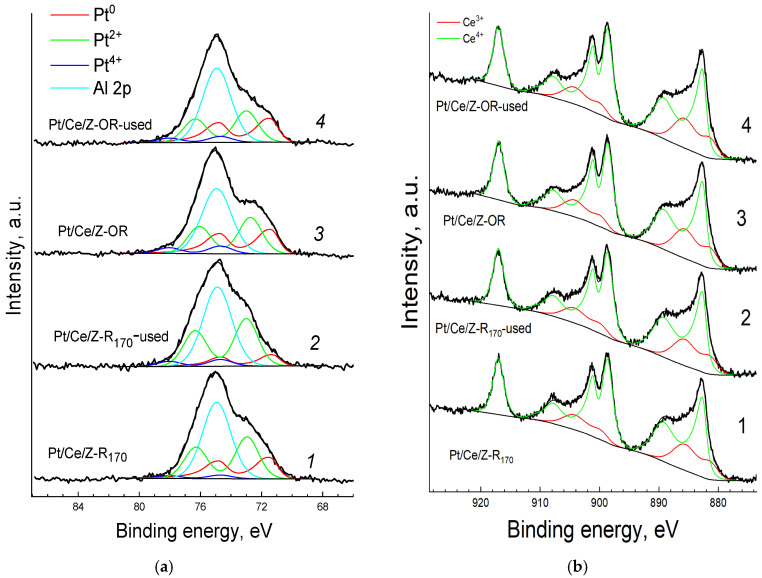
Pt4f (**a**) and Ce 3d (**b**) XPS-spectra of fresh (1, 3) and spent (2, 4) Pt/Ce/Z-R_170_ (1, 2) and Pt/Ce/Z-OR (3, 4) samples. Experimental spectra (thick black lines), fitted components (color lines), and resulting envelopes (thin black lines) are shown.

In the survey XPS spectra of the samples (Figure S2), only lines of cerium, platinum, oxygen, silicon, aluminum and adventitious carbon were observed. High-resolution Pt4f and Ce3d spectra of the fresh and spent catalysts are shown in Figure 7. The Pt4f_7/2_ spectra (Figure 7a) were fitted with a doublet of asymmetric components of metallic platinum (Pt^0^) and two doublets of symmetric components of oxidized species. The binding energies and percentages of components are summarized in Table 2. The binding energies of Pt4f_7/2_ components are within the range reported for Pt^0^ and two oxidized forms of Pt, Pt^2+^(PtO) and Pt^4+^ (PtO_2_) [66], but binding energies in the range of 72–75 eV may also be associated with the formation of other electron-deficient states of Pt in surface hydroxides or mixed oxides [25]. The assignment of these bands is ambiguous, but the presence of oxidized Pt^ox^ platinum in different forms of Pt^2+^ and Pt^2+δ^ on the surface of all the samples is well established. The Ce3d spectra (Figure 7b) have a complex shape due to the presence of shake-up satellites and the superposition of components of two oxidized forms of Ce: Ce^3+^ and Ce^4+^. These spectra were fitted with two synthetic components of Ce^3+^ and Ce^4+^ as described previously in [67].

The percentages of Pt and Ce in different electronic states on the surface of samples calculated from the XPS data are shown in Table 2. Modification with cerium changes the Pt^ox^/Pt^0^ ratio in the catalysts. The order of introduction of Pt and Ce affects the state of platinum in the catalyst. In the sample prepared by protocol A (Figure 1), when Ce was deposited to the Pt_170_/Z surface, only oxidized platinum is observed, and the platinum component in the Pt4f spectrum with the highest binding energy of 74.5 eV is strong, most likely due to interaction of platinum with cerium [33,46,47,48]. At the same time, in Pt/Ce/Z-R_170_ and Pt/Ce/Z-R_300_ composites produced by protocol B with the reverse order of component introduction followed by reduction in hydrogen at different temperatures, the percentage of metallic platinum increases and the ratio of oxidized forms of platinum changes. In all the samples, the less oxidized state of Pt with lower binding energy predominates. As can be seen from Table 2 and Figure 7, the percentage of Pt^2+δ^ with the highest binding energy (Eb = 74.5–74.7 eV) is higher in the sample that was subjected to oxidative–reductive treatment and reduced at an elevated temperature of 300 °C. The appearance of this form, in our opinion, is associated with the strengthening of the platinum interaction with cerium, as was suggested in [49,50,51]. Most likely, it is different from the electron deficient state with a close binding energy of 74.5 eV in monometallic samples caused by the interaction of platinum with the acidic centers of the zeolite [26].

Cerium in the fresh bimetallic samples is mainly (88–89%) in the form of Ce^4+^ (Table 2, short recording time (in parentheses)), but partial reduction of Ce^4+^ to Ce^3+^ takes place during the spectra acquisition. Moreover, in the samples produced by protocol A with the introduction of cerium cations after platinum (Ce/Pt_170_/Z), the reduction of Ce^4+^ under the action of the X-ray beam is more pronounced than in the samples synthesized by protocol B (Pt/Ce/Z-R_170_ and Pt/Ce/Z-R_300_). The percentage of Ce^3+^ increases in these samples by 31 and 11–12%, respectively (Table 2). Apparently, the deposition of platinum on the surface of zeolite previously modified with cerium cations results in a more efficient Pt-Ce interaction that stabilizes Ce^4+^ species.

### 2.4. Catalytic Performance in the CO Oxidation

**Figure 8:**
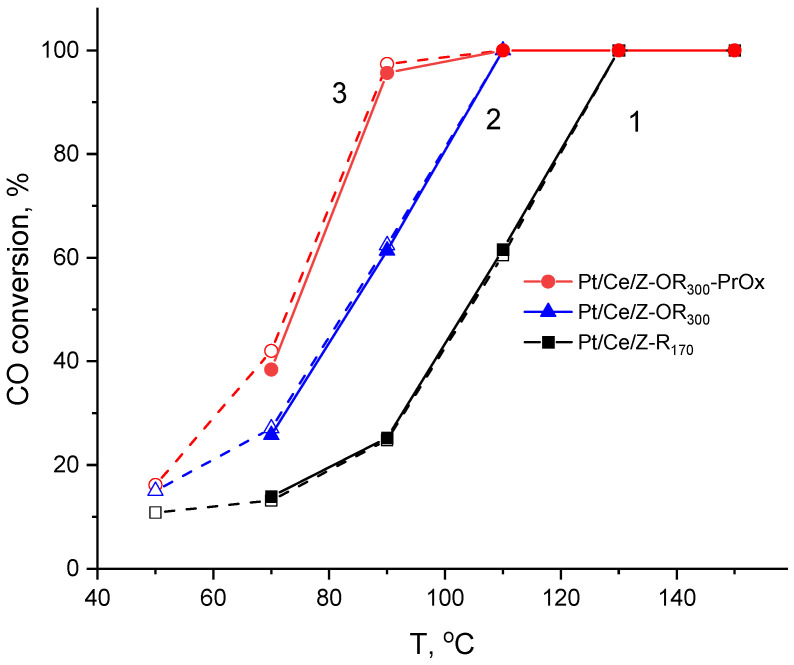
Temperature dependencies of the CO conversion in CO-TOX for PtCo-modified zeolites: 1—Pt/Ce/Z-R_170_, 2—Pt/Ce/Z-OR_300_, and 3—Pt/Ce/Z-OR_300_ tested in PROX.

The test results of the mono- and bimetallic PtCe-modified zeolites in CO-TOX and PROX reactions are compared in Table 3. Several temperature dependencies of the CO conversion are shown in Figure 8. Monometallic Pt/Z and Ce/Z zeolites demonstrate low activity in the oxidation of CO with oxygen, and the temperatures for 50% CO conversion (T_50_) are above 200 °C (Table 3). At the same time, for the bimetallic Pt-Ce composites, the T_50_ values are almost 2 times lower and range from 70 to 110 °C depending on the composition and treatment of the catalyst. The scale of the synergistic effect of platinum and cerium depends primarily on the order of introduction of these components. In the case of the synthesis of a bimetallic composite by protocol A (Figure 1), when Pt is introduced first and undergoes mild reduction at 170 °C, the Ce/Pt_170_/Z catalyst immediately turns out to be quite active: T_50_ has the lowest value of 80 °C, and 100% CO conversion is already achieved at 120 °C. Additional reduction of the catalyst at 300 °C only deteriorates its performance (CePt_170_/Z-R_300_ sample) (Table 3).

When Ce was deposited before Pt (Figure 1, protocol B), and the resulting sample was subjected to reduction with hydrogen at different temperatures, the catalyst reduced under milder conditions (Pt/Ce/Z-R_170_) turns out to be more active than the catalyst reduced at 300 °C (Pt/Ce/Z-R_300_); the T_50_ values are 105 and 115 °C, respectively (Table 3). However, the activity of these catalysts can be improved by additional redox treatments according to the Scheme depicted in Figure 1. As can be seen from Figure 8 (curves 1 and 2), the CO oxidation over the resulting Pt/Ce/Z-OR_300_ led to a 100% conversion at 110 °C compared to 130 °C for Pt/Ce/Z-R_170_.

The oxidation of CO in the H_2_ excess (CO-PROX) proceeds at lower temperatures; however, when certain temperatures are reached, the CO conversion decreases due to a side reaction of hydrogen oxidation. The maximum CO conversions (X_max_) and the temperatures at which they were achieved (T_max_) are summarized in Table 3. Nevertheless, it can be said that the co-action of Pt and Ce is also manifested under CO-PROX conditions. Although 100% CO conversion is achieved on monometallic Pt/Z catalyst, this requires a high temperature of 190 °C. At the same time, on the most active catalysts in CO-TOX (Ce/Pt_170_/Z and Pt/Ce/Z-OR_300_), maximum CO conversions (80–90%) are achieved at a significantly lower temperature of 90 °C. Apparently, these catalysts are also highly active in the oxidation of hydrogen, which reduces the selectivity of the process due to the consumption of oxygen for both competing reactions.

In addition, the spent catalysts after CO-PROX reaction demonstrated improved catalytic properties in repeated CO-TOX tests. A comparison of curves 2 and 3 (Figure 8) shows that the T_100_ temperature under CO-TOX conditions decreases for Pt/Ce/Z-OR_300_ catalyst by 15 degrees (from 110 to 95 °C). A similar decrease in the T_100_ temperature by 10–20 °C after catalytic tests under CO-PROX conditions was found for the other catalysts listed in Table 3. This effect is probably due to the promoting action of H_2_ on the catalytic performance of the Pt/CeO_2_ systems [46]. As can be seen from Table 3, it was not observed for the Pt/Z catalysts.

## 3. Discussion

The experimental results revealed a strong dependence of the catalytic properties of PtCe-modified zeolites on the order of components introduction and post-synthetic treatment conditions (temperature; oxidizing or reducing atmosphere). In all cases, the introduction of cerium enhances the catalytic behavior of platinum, which is associated with the role of CeO_2_ in the oxidation process as an additional source of oxygen [19,40,41,42]. Indeed, the XPS analysis demonstrated mainly a Ce^4+^ oxidation state of Ce in all studied bimetallic catalysts (Table 3). It is well known that the interaction of Pt and Ce at the interface with the formation of new active Pt-O-Ce species plays a key role in ensuring the synergistic properties of PtCe systems [33,48,49]. Apparently, the conditions of sample synthesis, primarily the sequence of component deposition, strongly influence their interaction and interface formation. For samples prepared by protocol A (Figure 1), when Pt was introduced before Ce, platinum exists predominantly (73%) in an electron-deficient Pt^2+δ^ state with the highest binding energy of the Pt4f_7/2_ component being 74.7 eV (Table 2). Initially, there are no metallic Pt^0^ species on the surface of the fresh sample. However, under the process conditions, such charged single atoms can easily transform into small clusters that are active in catalysis, but they are difficult to detect. This explanation for the improved activity of the supported Pt/CeO_2_ catalysts with a close platinum content of 0.25 wt% was proposed previously [24]. Moreover, samples with higher platinum loadings of 0.5–0.7 wt%, including nanosized platinum oxides, were less active. However, it should be noted that even for the most active samples with the optimal platinum loading of 0.25%, 100% CO conversion was achieved only at temperatures around 200 °C. The PtCe zeolites prepared in our work provide complete CO conversion at significantly lower temperatures of 95–110 °C (Table 3).

The reverse order of components’ deposition on the zeolite surface by protocol B (Figure 1) led to lower initial activities of the Pt/Ce/Z catalysts. The T_100_ values in this case were 130–140 °C depending on a reduction temperature of 170 or 300 °C, but this is also lower than the values reported for similar catalysts in most works [17,18,23,24]. Moreover, the activity of the Pt/Ce/Z catalysts was improved after additional thermal treatment initially in the oxidizing and then in the reducing atmosphere (Figure 1, Scheme).

In contrast to Ce/Pt/Z catalysts prepared by protocol A, Pt/Ce-zeolites with the reverse sequence of components’ introduction (protocol B) contained platinum in three electronic states (Table 2): along with the oxidized states of platinum, represented by Pt^2+^ and Pt^2+δ^ species, the percentage of metallic Pt^0^ was significant (40%). It is important to note that redox treatment increases the percentage of Pt^2+δ^ species that are apparently responsible for the interaction of platinum with cerium: in the Pt/Ce/Z-OR_300_ sample, its percentage is 2 times higher than in Pt/Ce/Z-R_170_. As a result, the activity of the Pt/Ce/Z-OR_300_ catalyst significantly increases, and the complete CO conversation is achieved at 110 °C. When the heating–cooling cycle is repeated, the catalysts demonstrate stable activity in at least four consecutive TOX-CO reaction cycles. According to the XPS data, the electronic states of platinum and cerium in the spent Pt/Ce/Z-OR_300_ catalyst after several cyclic tests in the CO-TOX reaction and in the fresh one are practically the same (Table 2). Similar catalytic tests of the less active Pt/Ce/Z-R_170_ sample increase the percentages of Pt^2+^ species with a lower binding energy of the Pt4f_7/2_ component of about 73 eV. This state most likely corresponds to the formation of a low-activity surface PtO oxide; at the same time, the percentage of metallic Pt^0^ decreases. Thus, it can be concluded that the presence of a metallic state is also important for ensuring high activity since it provides the necessary adsorption CO sites. The interaction of Pt and Ce and the formation of the Pt-O-Ce interface ensures high oxidation efficiency due to the participation of lattice oxygen and cerium oxide vacancies [19,49,50,51].

It is also important to note the different state of cerium in the samples prepared by protocols A and B. Its reducibility under the X-ray beam during XPS analysis varies significantly (Table 2). In the monometallic Ce/ZSM-5 sample containing 2–4% Ce [65], cerium is in the form of cations or oxocations of Ce^3+^ or Ce^4+^ in zeolite channels, as well as in the form of oxide CeO_2_ particles on the surface. Therefore, when introducing platinum according to protocol B onto the surface of the Ce/Z zeolite containing Ce^3+^ or Ce^4+^, the interaction of cationic forms of cerium with the oxidized platinum species from H_2_PtCl_6_ is accompanied by an increase in the percentage of Ce^4+^ species and an increase in the percentage of reduced Pt^0^ species compared to the monometallic Ce/Z and Pt_170_/Z samples. The corresponding changes were observed in the XPS spectra of the Pt/Ce/Z-R_170_ and Pt/Ce/Z-R_300_ samples.

The IR- spectrum of adsorbed CO on the bimetallic Pt/Ce/Z-R_170_ sample (Figure 2) confirms the presence of cationic forms of cerium in ion-exchange positions in the zeolite channels (absorption band at 2187–2189 cm^−1^), as well as in the form of oxides (band at around 2170 cm^−1^). The presence of cerium oxides in these catalysts is confirmed by the TEM (Figure 6) and XRD (Figure S1) data. Furthermore, the absence of platinum nanoparticles on the surface of the bimetallic sample according to the DRIFT-spectroscopy (Figure 2) and TEM (Figure 6) data also indicates the formation of an interfacial Pt-O-Ce band.

In the case of protocol A, when cerium was introduced onto the Pt_170_/Z zeolite surface containing partially reduced platinum (Table 2), the interaction of the two metals when heated in an air flow should lead to the oxidation of platinum and an increase in the percentage of Ce^3+^ species due to the electron transfer from Pt^0^ to cerium Ce^4+^. However, the resulting electronic state of cerium is not stable enough, and Ce^4+^ readily transforms into Ce^3+^ under the X-ray beam during XPS analysis. Apparently, the interaction of platinum with the cationic forms of cerium located in ion-exchange positions (protocol B) is more efficient than in the case of the reverse order of components’ deposition.

Finally, it should be emphasized that the catalysts initially tested in the H_2_ excess in CO-PROX become more active in the TOX reaction. The T_100_ values (Table 3) for all the bimetallic catalysts decrease by approximately 10–20 °C during repeated CO-TOX tests. This effect is absent for the monometallic Pt/Z sample. This PROX-promoted activity in the CO oxidation cannot be due to Pt reduction. As can be seen from Table 3, the additional Ce/Pt_170_/Z catalyst reduction at 300 °C only worsens catalytic properties.

The promoting effect observed only on bimetallic composites may be related to surface- and water-mediated mechanisms of CO oxidation that are well known for PtCe catalysts of different types [52,53,54]. In both mechanisms, the promoting effect of hydrogen or water was due to the production of OH groups near the Pt-O-Ce interface. There is no such effect without the interfacial Pt-O-Ce bond formation on monometallic Pt catalysts. The following reasons may be responsible for promotion effects: (i) the appearance of highly active Pt^δ+^ species in interaction with ceria; (ii) the formation of mobile and active OH intermediates or the creation of oxygen vacancies that increases oxygen mobility; (iii) reaction of hydroxyl with adsorbed CO to form COOH intermediates producing CO_2_ and H_2_O. The mechanism of the CO oxidation strongly depends on the specific catalyst system. In accordance with experimental data and DFT calculations [54] surface-active oxygen species captured from O_2_ on zeolite-supported PtCe catalysts play a crucial role in the CO oxidation.

Thus, the results of this work demonstrate the possibilities of synthesis of efficient catalysts with a reduced platinum content based on zeolite ZSM-5, the structural features of which provide favorable conditions for the metal-oxide interaction. Additional thermal treatments under various conditions, including the reaction atmosphere of the PROX process, allowed for further improvement in the activity of PtCe-modified zeolites in the CO oxidation. The complete CO oxidation over 0.2 wt% Pt catalysts was achieved at an extremely low temperature of 95 °C. A similarly low value of 90 °C has been reported for hollow Pt/CeO_2_ nanocatalysts with a higher Pt loading of 3 wt% [52].

A similar strategy for the formation of active catalysts for CO oxidation based on Pt^2+^, small Pt nanoparticles, and Ti-modified zeolite was proposed earlier [68]. However, in this case, the T_90_ value for the catalyst with a similar platinum loading of 0.2 wt% was 175 °C, which is significantly higher than in our work. Another zeolite-based PtCe catalyst with an ultra-low platinum loading of 0.1 wt% allowed for the complete oxidation of CO at a temperature of about 150 °C [54]. In the authors opinion, the highly dispersed Pt-CeO_2_ interfaces and water promotion effect were the reasons for the high catalytic performance. The data of this work and the examples provided show that the use of zeolites and special approaches to improve the Pt-CeO_2_ interaction with an active Pt-O-Ce interface formation seems to be a promising way to reduce the content of precious metals in catalysts for the low-temperature oxidation of CO and other hazardous pollutants.

## 4. Materials and Methods

### 4.1. Catalyst Synthesis

The HZSM-5 (HZ) was prepared from NH_4_ZSM-5 zeolite (“Zeolyst”, 425 m^2^/g, SiO_2_/Al_2_O_3_ = 55) by calcination at 550 °C in an air flow for 8 h. The monometallic Pt/Z and Ce/Z samples were synthesized by incipient wetness impregnation of HZ with aqueous solutions of H_2_PtCl_6_ (40% Pt, Sigma-Aldrich, Saint Louis, MO, USA) and Ce(NO_3_)_3_ (Sigma-Aldrich), respectively, followed by drying at 120 °C for 8 h and calcination in an air flow at 500 °C for 3 h. Pt/Z was additionally reduced by H_2_ at 170 °C for 3 h. Bimetallic Pt-Ce-modified zeolites were synthesized by sequential impregnation, using different orders of metal deposition (Figure 1). In the first approach (path A), initially platinum was deposited from an aqueous solution of H_2_PtCl_6_, dried at room temperature and at 120 °C for 8 h, and then reduced in a H_2_ flow at 170 °C. The reduction temperature is denoted as a subscript number in the sample name (Pt_170_/Z). In the next step, cerium was deposited from an aqueous solution of Ce(NO_3_)_3_, followed by drying and calcination at 120 °C for 8 h and in an air flow at 500 °C for 3 h. The sample was denoted as Ce/Pt_170_/Z. The first element symbol in the sample name designates the last deposited metal.

In the second approach (path B), cerium was deposited first, dried at room temperature and at 120 °C (8 h), and then platinum was deposited, dried at 120 °C for 8 h, and calcined at 500 °C for 3 h in an air flow. The Pt/Ce/Z samples were then reduced in a flow of H_2_ at temperatures of 170 or 300 °C to yield the Pt/Ce/Z-R_170_ or Pt/Ce/Z-R_300_ catalysts. The Pt/Ce/Z-R_170_ samples were further subjected to oxidative–reductive treatments in an air flow at 500 °C and then in a hydrogen flow at 300 °C. These samples were designated as Pt/Ce/Z-OR_300_. According to the atomic absorption and atomic emission spectroscopy data, the Pt and Ce contents in all samples were close to 0.2 and 4.0 wt.%, respectively. Catalyst synthesis routes and post-synthetic treatments were shown schematically in Figure 1.

### 4.2. Catalyst Characterization

Pt and Ce contents were measured by atomic absorption spectroscopy on a Thermo ICE-3000 spectrometer (Thermo Fisher Scientific Inc., Waltham, MA, USA) and by inductively coupled plasma (ICP) atomic emission spectroscopy using an iCAP 6300 Duo spectrometer (Thermo Fisher Scientific, USA). The measured concentrations agreed well with the calculated values.

The phase composition of the samples was analyzed by powder X-ray diffraction (XRD) analysis using a STADI-P, STOE GmbH, Darmstadt, Germany (CuKα radiation). The XRD patterns were recorded in a 2θ range of 10–40 degrees.

Scanning electron microscopy (SEM) images of the catalyst surfaces were recorded on a JSM-6000 NeoScope scanning electron microscope (“JEOL”, Peabody, MA, Japan) with a JEOL JED-2300 Analysis Station Plus EDX system in the high vacuum mode with an accelerating voltage of 10–15 kV. The signal detection mode was SEI (secondary electron imaging).

Transmission electron microscopy (TEM) analysis was performed on a JEM 2100F/UHR microscope (JEOL, Japan) with a resolution of 0.2 nm. The elemental compositions of the samples were determined by energy-dispersive X-ray (EDX) analysis using a JED-2300 accessory (JEOL, Japan). The sample preparations for the TEM study and image processing were described in detail in [39]. Interplanar distances (d) were determined using the ImageJ 1.47 program (URL https://imagej.nih.gov/ĳ/download.html) (accessed on 25 December 2025). The lattice d-spacing values were calculated from the fast Fourier transformation (FFT) patterns for planes visible in high-resolution TEM images. The crystal structures of the ordered atomic domains were identified using the ICDD data base (https://www.icdd.com).

The surface composition and electronic state of the metals were analyzed by XPS using an Axis Ultra DLD spectrometer (Kratos Analytical, Stretford, UK). Pass energies of 160 and 40 eV were used, respectively, for survey spectra and high-resolution scans. Ce^4+^ species on the surface are known to partly reduce under X-ray beams during XPS analysis. To monitor and eliminate this reduction, a fast scan (acquisition time of several minutes) high-resolution Ce3d spectrum was recorded just after the X-ray source was switched on. Accumulation of high-resolution spectra with a reliable signal-to-noise ratio (including Ce3d spectrum with longer acquisition time) required 1–2 h. The spectra were charge-referenced to the Si 2p binding energy of 103.6 eV, typical of silicon oxides. The Pt4f spectra were fitted with three doublets: one associated with metallic and two with oxidized Pt species taking into account the overlapping of Pt4f and Al2p lines as described in [25]. The Ce3d spectra were fitted with the synthetic Ce^3+^ and Ce^4+^ components of complex shape as described previously in [67].

Diffuse reflectance infrared Fourier transform spectroscopy (DRIFTS) analysis of adsorbed CO was performed on an Infralum FT-801 Fourier spectrometer (Lyumeks–Sibir’, Novosibirsk, Russia) equipped with a diffuse reflection attachment in the 900–6000 cm^−1^ range (4 cm^−1^ resolution, 256 scans). Catalyst pellets were placed in a quartz tube with the CaF_2_ optical window and heated under vacuum at a rate of 10 °C/min to 400 °C and then kept at this temperature for 100 min. Spectra of the adsorbed CO were recorded at room temperature at equilibrium gas pressure varied from 0.1 to 4.4 kPa as well as at a residual pressure of 0.05 kPa after evacuation. The IR spectra were transformed into the Kubelka–Munk function as described in [38].

### 4.3. Catalytic Tests

CO oxidation was performed in a quartz fixed-bed flow reactor operated at atmospheric pressure at temperatures of 50–250 °C in heating–cooling modes as described in [26,39]. The temperature was changed in steps of 20 °C; each temperature was maintained for 20 min and cyclic tests were repeated several times. The gas mixtures of 1 vol.% CO, 1 vol.% O_2_, 98 vol.% He and 1 vol.% CO, 1 vol.% O_2_, 49 vol.% H_2_, and 49 vol.% He were used for CO-TOX and PROX tests. A total of 250 mg of catalyst (grain size 0.4–0.6 mm) with an equivalent amount of quartz sand was placed in the reactor and heated for 1 h at 350 °C in a helium flow. The flow rate was 10 mL/min. The experimental conditions were chosen in accordance with earlier studies [38]. The reaction mixture was analyzed on-line by GC using a Crystal 2000 chromatograph (Chromatec SDO JSC, Yoshkar-Ola, Russia) equipped with a thermal conductivity detector. CO-PROX resulted in only CO_2_ and H_2_O formation; no methane was detected. To estimate the catalytic activity, the temperature dependencies of the steady-state CO conversion modes were analyzed. The following values were used to compare catalyst performances: CO conversion at 110 °C (X_110_,%), T_50_ and T_100_ temperatures of 50 and 100% CO conversions in CO –TOX, the maximum CO conversion (X_max_,%) reached in CO-PROX, and the corresponding temperature T_max_. The data presented are obtained as a result of reproducible experiments.

## 5. Conclusions

PtCe-catalysts with low Pt loading of 0.2 wt% were prepared by impregnation of the protonic form of ZSM-5 zeolite (Z) with aqueous solutions of H_2_PtCl_6_ and Ce(NO_3_)_3_ with different impregnation sequences. The best catalytic properties were found when Pt was deposited before cerium and reduced at 170 °C; the total CO oxidation for Ce/Pt_170_/Z catalyst was achieved at 120 °C. The reverse sequence of metal deposition (Pt/Ce/Z sample) resulted in a less active fresh catalyst, but after two-step redox treatment, its activity strongly improved, and the temperature of 100% CO conversion dropped to 110 °C. In contrast to monometallic Pt/Z catalyst, activity of PtCe-zeolites was further improved after testing in mixtures with H_2_ excess (CO-PROX). Such samples, when tested repeatedly in the absence of hydrogen (CO-TOX), provided 100% CO conversion at 95 °C. This effect can be associated with the promoting action of hydrogen and water that resulted in a new reaction pathway with the participation of the OH–group in the CO oxidation [46].

A significant improvement in the catalytic activity of fresh PtCe-modified zeolites after redox (OR) processing and testing in H_2_ excess is associated, in our opinion, with the structural changes in the catalyst that lead to the formation of new active sites for CO oxidation at the interfacial Pt-O-Ce bonds. The best Pt/Ce/Z-OR_300_ catalyst contains platinum species in different electronic states and a stable interfacial Pt-O-Ce bond, these are key factors responsible for the catalyst efficiency. The ratios of Pt^Ox^/Pt^0^ on the surface were the same (about 1.5) for the fresh and spent samples, and the high-performance of this catalyst remained stable during repeated tests.

As far as we know, the best low-percentage (0.1–0.2 wt.%) Pt zeolite-supported catalysts provide 100% CO conversion at temperatures of 150 °C and above [54,68]. For most catalysts reported in the literature [17,18,19,20,21], a complete CO conversion can be achieved at temperatures below 150 °C only for Pt loadings above 1%. Thus, the strategy of synthesis and activation of low-loaded PtCe catalysts proposed in this work can be useful for designing advanced zeolite-based noble metal catalysts for ecologically important combustion of CO, hydrocarbons, and other volatile organic compounds.

## Figures and Tables

**Table 1:** Surface composition of catalysts measured by SEM-EDX.

Catalyst	At.%				
	Pt	Ce	Si	Al	O
Pt_170_/Z	0.02	-	26.05	0.98	72.95
Ce/Z	-	0.53	27.80	1.03	70.64
Pt/Ce/Z-R_170_	0.02	0.53	27.19	1.02	71.24
Ce/Pt_170_/Z	0.01	0.55	28.78	1.09	69.57

**Table 2:** Binding energies of Pt4f_7/2_ and Ce3d component and their percentages in the XPS spectra of mono- and bimetallic PtCe-Z catalysts.

E_b_, eV	71.4–71.5	72.7–72.9	74.5–74.7	881.8	882.9
At. %	Pt			Ce *	
Catalyst	Pt^0^	Pt^2+^	Pt^2+δ^	Ce^3+^	Ce^4+^
Ce/Z	-	-	-	51	49
Pt_170_/Z	28	22	50	-	-
Ce/Pt_170_/Z	0	27	73	42 (11)	58 (89)
Pt/Ce/Z-R_170_	35	60	5	22 (11)	78 (89)
Pt/Ce/Z-R_170_-spent	18	72	10	22 (13)	78 (87)
Pt/Ce/Z-OR_300_	38	51	11	23 (11)	77 (89)
Pt/Ce/Z-OR_300_-spent	42	49	9	23 (12)	77 (88)

* Data for fast acquisition of Ce3d spectrum are shown in parentheses.

**Table 3:** CO conversions at 110 °C (X_110_) and T_50_ and T_100_ for catalysts tested in CO-TOX and maximum CO conversions (X_max_) and temperatures of maximum conversion (T_max_) in CO-PROX.

CO Oxidation	TOX				PROX	
Catalyst	X_110_, %	T_50_, °C	T_100_, °C	T *_100_, °C	T_max_, °C	X_max_, %
Ce/Z	1	250	>290	-	-	-
Pt_170_/Z	12	200	210	210	190	100
PtCe/Z-R_170_	62	105	130	110	110	65
PtCe/Z-R_300_	45	115	140	130	110	56
PtCe/Z-OR_300_	100	80	110	95	90	82
CePt_170_/Z	97	80	120	95	90	91
CePt_170_/Z-R_300_	-	-	170	-	130	17

* achieved after PROX.

## Data Availability

Data are contained within the article and Supplementary Materials.

## Supplementary Materials

The following supporting information can be downloaded at https://www.mdpi.com/article/10.3390/molecules31010156/s1: Figure S1. X-ray diffraction patterns of initial ZSM-5 zeolite (Z) and bimetallic Pt/Ce/Z sample; Figure S2. Survey XPS spectra of Pt/Ce/Z-catalysts.

## References

[1] Jeong H., Kwon O., Kim B.S., Bae J., Shin S., Kim H.E., Kim J., Lee H. (2020). Highly durable metal ensemble catalysts with full dispersion for automotive applications beyond single-atom catalysts. Nat. Catal..

[2] Jing P., Gong X., Liu B., Zhang J. (2020). Recent advances in synergistic effect promoted catalysts for preferential oxidation of carbon monoxide. Catal. Sci. Technol..

[3] Zheng F., Zhang W., Guo Q., Yu B., Wang D., Chen W. (2024). Metal clusters confined in porous nanostructures: Synthesis, properties and applications in energy catalysis. Coord. Chem. Rev..

[4] Freund H.-J., Meijer G., Scheffler M., Schlögl R., Wolf M. (2011). CO oxidation as a prototypical reaction for heterogeneous processes. Angew. Chem. Int. Ed..

[5] Neumann S., Gutmann T., Buntkowsky G., Paul S., Thiele G., Sievers H., Bäumer M., Kunz S. (2019). Insights into the reaction mechanism and particle size effects of CO oxidation over supported Pt nanoparticle catalysts. J. Catal..

[6] Liu J., Hensley A.J.R., Giannakakis G., Therrien A.J., Sukkar A., Schilling A.C., Groden K., Ulumuddin N., Hannagan R.T., Ouyang M. (2021). Developing single-site Pt catalysts for the preferential oxidation of CO: A surface science and first principles-guided approach. Appl. Catal. B.

[7] Khan H.A., Abou-Daher M., de Freitas A.L.S., Subburaj J., Tall O.E.I., Farooq A. (2024). Performance studies of Pt, Pd and PtPd supported on SBA-15 for wet CO and hydrocarbon oxidation. Catal. Today.

[8] Chen Y., Zhao J., Zhao X., Wu D., Zhang N., Du J., Zeng J., Li X., Salmeron M., Liu J. (2025). Stabilizing supported atom-precise low-nuclearity platinum cluster catalysts by nanoscale confinement. Nat. Chem. Eng..

[9] Chen J., Wu Y., Hu W., Qu P., Zhang G., Granger P., Zhong L., Chen Y. (2020). New insights into the role of Pd-Ce interface for methane activation on monolithic supported Pd catalysts: A step forward the development of novel PGM Three-Way Catalysts for natural gas fueled engines. Appl. Catal. B.

[10] Danielis M., Colussi S., De Leitenburg C., Soler L., Llorca J., Trovarelli A. (2018). Outstanding Methane Oxidation Performance of Palladium-Embedded Ceria Catalysts Prepared by a One-Step Dry Ball-Milling Method. Angew. Chem..

[11] Lv Y., Guo J., Ding C., Yan Y., Chen H., Ma L., Wang J., Meng Y., Ma Z., Liu P. (2023). Highly dispersed Pt clusters within ZSM-5 stabilized by alkali metal ions and Al sites for partial methane oxidation. Mol. Catal..

[12] Jiang Z., Chen D., Deng W., Guo L. (2022). Different morphological ZSM-5 zeolites supported Pt catalysts for toluene catalytic combustion. Chem. Phys. Impact.

[13] Boronin A.I., Slavinskaya E.M., Figueroba A., Stadnichenko A.I., Kardash Y.Y., Stonkus O.A., Fedorova E.A., Muravev V.V., Svetlichnyi V.A., Bruix A. (2021). CO oxidation activity of Pt/CeO2 catalysts below 0 ◦C: Platinum loading effects. Appl. Catal. B Environ..

[14] Lin J., Wang X., Zhang T. (2016). Recent progress in CO oxidation over Pt-group-metal catalysts at low temperatures. Chin. J. Catal..

[15] Chen Y., Lin J. (2023). Design of efficient noble metal single-atom and cluster catalysts toward low-temperature preferential oxidation of CO in H_2_. Int. J. Hydrogen Energy.

[16] Daniel S., Monguen C.K.F., Ayodele O.B., Tian Z.Y. (2023). Tailored synthesized Pt/ZSM-5 catalysts with excellent water vapor stability for low temperature oxidation of CO and C_3_H_6_. J. Environ. Chem. Eng..

[17] Slavinskaya E.M., Stadnichenko A.I., Domínguez J.E.Q., Stonkus O.A., Vorokhta M., Šmíd B., Castro-Latorre P., Bruix A., Neyman K.M., Boronin A.I. (2023). States of Pt/CeO_2_ catalysts for CO oxidation below room temperature. J. Catal..

[18] Jiang B., Cha X., Huang Z., Hu S., Xu K., Cai D., Xiao J., Zhan G. (2022). Green fabrication of hierarchically-structured Pt/bio-CeO_2_ nanocatalysts using natural pollen templates for low-temperature CO oxidation. Mol. Catal..

[19] Nie L., Mei D., Xiong H., Peng B., Ren Z., Hernandez X.I.P., DeLaRiva A., Wang M., Engelhard M.H., Kovarik L. (2017). Activation of surface lattice oxygen in single-atom Pt/CeO_2_ for low-temperature CO oxidation. Science.

[20] Gatla S., Aubert D., Flaud V., Grosjean R., Lunkenbein T., Mathon O., Pascarelli S., Kaper H. (2019). Facile synthesis of high-surface area platinum-doped ceria for low temperature CO oxidation. Catal. Today.

[21] Liu Z., Liu K., Yang X., Chen X., Shen X., Li Y., Fang Y., Liu Y., Zhao J., Yang X. (2024). In-situ formed stable Pt nanoclusters on ceria-zirconia solid solutions induced by hydrothermal aging for efficient low-temperature CO oxidation. Chem. Eng. J..

[22] Feng C., Liu X., Zhu T., Hu Y., Tian M. (2021). Catalytic oxidation of CO over Pt/TiO_2_ with low Pt loading: The effect of H_2_O and SO_2_. Appl. Catal. A.

[23] Hatanakaa M., Takahashia N., Tanabea T., Nagaia Y., Dohmaea K., Aokib Y., Yoshidab T., Shinjoha H. (2010). Ideal Pt loading for a Pt/CeO_2_-based catalyst stabilized by a Pt-O-Ce bond. Appl. Catal. B.

[24] Dong J., Zhang Y., Li D., Adogwa A., Huang S., Yang M., Yang J., Jin Q. (2023). Reaction-driven evolutions of Pt states over Pt-CeO_2_ catalysts during CO oxidation. Appl. Catal. B.

[25] Golubina E.V., Rostovshchikova T.N., Lokteva E.S., Maslakov K.I., Nikolaev S.A., Shilina M.I., Gurevich S.A., Kozhevin V.M., Yavsin D.A., Slavinskaya E.M. (2021). Role of surface coverage of alumina with Pt nanoparticles deposited by laser electrodispersion in catalytic CO oxidation. Appl. Surf. Sci..

[26] Shilina M.I., Krotova I.N., Maksimov S.V., Maslakov K.I., Nikolaev S.A., Udalova O.V., Gurevich S.A., Yavsin D.A., Rostovshchikova T.N. (2023). Total and preferential CO oxidation on low-loaded Pt-HZSM-5 zeolites modified using laser electrodispersion. Russ. Chem. Bull..

[27] Chen Y., Wan Q., Cao L., Gao Z., Lin J., Li L., Pan X., Lin S., Wang X., Zhang T. (2022). Facet-dependent electronic state of Pt single atoms anchoring on CeO_2_ nanocrystal for CO (preferential) oxidation. J. Catal..

[28] Daelman N., Capdevila-Cortada M., Lypez N. (2019). Dynamic charge and oxidation state of Pt/CeO_2_ single-atom catalysts. Nat. Mater..

[29] Wang W., Li D., Yu H., Liu C., Tang C., Chen J., Lu J., Luo M. (2021). Insights into Different Reaction Behaviors of Propane and CO Oxidation over Pt/CeO_2_ and Pt/Nb_2_O_5_: The Crucial Roles of Support Properties. J. Phys. Chem. C.

[30] Wang S., Wang S., Zong X., Wang S., Dong X. (2023). CO oxidation with Pt catalysts supported on different supports: A comparison of their sulfur tolerance properties. Appl. Catal. A.

[31] Park D., Kim S.M., Kim S.H., Yun J.Y., Park J.Y. (2014). Support effect on the catalytic activity of two-dimensional Pt nanoparticle arrays on oxide substrates. Appl. Catal. A.

[32] An K., Alayoglu S., Musselwhite N., Plamthottam S., Melaet G., Lindeman A.E., Somorjai G.A. (2013). Enhanced CO Oxidation Rates at the Interface of Mesoporous Oxides and Pt Nanoparticles. J. Am. Chem. Soc..

[33] Piconen A., Riva M., Brambilla A., Calloni A., Bussetti G., Finazzi M., Ciccacci F., Duò L. (2016). Reactive metal–oxide interfaces: A microscopic view. Surf. Sci. Rep..

[34] Tian X., Shan Y., Zhang J., Yan Z., Sun Y., Ding W., Yu Y. (2023). The study of Pt/zeolites for CO oxidation: Effects of skeleton structure and Si/Al ratio. Catal. Commun..

[35] Wang J., Guo X., Shi Y., Zhou R. (2021). Synergistic effect of Pt nanoparticles and micro-mesoporous ZSM-5 in VOCs low-temperature removal. J. Environ. Sci..

[36] Rostovshchikova T.N., Nikolaev S.A., Krotova I.N., Maslakov K.I., Udalova O.V., Gurevich S.A., Yavsin D.A., Shilina M.I. (2022). ZSM-5 and BEA zeolites modified with Pd nanoparticles by laser electrodispersion. The structure and catalytic activity in CO and CH_4_ oxidation. Russ. Chem. Bull..

[37] Kong F., Li G., Wang J., Shi Y., Zhou R. (2022). Promoting effect of acid sites in hierarchical porous Pt/ZSM-5 catalysts for low-temperature removal of VOCs. Appl. Surf. Sci..

[38] Shilina M., Krotova I., Nikolaev S., Cherkashina N., Stolarov I., Udalova O., Maksimov S., Rostovshchikova T. (2024). Advanced PtCo Catalysts Based on Platinum Acetate Blue for the Preferential CO Oxidation in H_2_-Rich Mixture. Catalysts.

[39] Shilina M., Krotova I., Nikolaev S., Gurevich S., Yavsin D., Udalova O., Rostovshchikova T. (2023). Highly Effective Pt-Co/ZSM-5 Catalysts with Low Pt Loading for Preferential CO Oxidation in H_2_-Rich Mixture. Hydrogen.

[40] Melchionna M., Fornasiero P. (2024). The role of ceria/precious metal interfaces in catalysis. RSC Appl. Interfaces.

[41] Montini M., Melchionna M., Monai P., Fornasiero P. (2016). Fundamentals and Catalytic Applications of CeO_2_ Based Materials. Chem. Rev..

[42] Chen S., Zhang K., Chen Y., Shao B., Zeng C., Yuan W., Yang H., Han Z.K., Jiang Y., Zhang Z. (2025). Interface engineering to regulate oxidation dynamics of supported nanoparticles. Nat. Commun..

[43] Nguyen T.S., Morfin F., Aouine M., Bosselet F., Rousset J.L., Piccolo L. (2015). Trends in the CO oxidation and PROX performances of the platinum-group metals supported on ceria. Catal. Today.

[44] Lykhach Y., Bruix A., Fabris S., Potin V., Matolínová I., Matolín V., Libuda J., Neyman K.M. (2017). Oxide-based nanomaterials for fuel cell catalysis: The interplay between supported single Pt atoms and particles. Catal. Sci. Technol..

[45] Neyman K.M., Kozlov S.M. (2022). Quantifying interactions on interfaces between metal particles and oxide supports in catalytic nanomaterials. NPG Asia Mater..

[46] Morfin F., Nguyen T.S., Rousset J.L., Piccolo L. (2016). Synergy between hydrogen and ceria in Pt-catalyzed CO oxidation: An investigation on Pt-CeO_2_ catalysts synthesized by solution combustion. Appl. Catal. B.

[47] Lee J., Ryou Y.S., Chan X., Kim T.J., Kim D.H. (2016). How Pt Interacts with CeO_2_ under the Reducing and Oxidizing Environments at Elevated Temperature: The Origin of Improved Thermal Stability of Pt/CeO_2_ Compared to CeO_2_. J. Phys. Chem. C.

[48] Vincent J.L., Crozier P.A. (2021). Atomic level fluxional behavior and activity of CeO_2_-supported Pt catalysts for CO oxidation. Nat. Commun..

[49] Kottwitz M., Li Y., Palomino R.M., Liu Z., Wang G., Wu Q., Huang J., Timoshenko J., Senanayake S.D., Balasubramanian M. (2019). Local Structure and Electronic State of Atomically Dispersed Pt Supported on Nanosized CeO_2_. ACS Catal..

[50] Kauppinen M.M., Daelman N., López N., Honkala K. (2023). The role of polaronic states in the enhancement of CO oxidation by single-atom Pt/CeO_2_. J. Catal..

[51] Lashina E.A., Slavinskaya E.M., Stonkus O.A., Stadnichenko A.I., Romanenko A.V., Boronin A.I. (2023). The role of ionic and cluster active centers of Pt/CeO_2_ catalysts in CO oxidation. Experimental study and mathematical modeling. Chem. Eng. Sci..

[52] Huang M., He J., Xu K., Cai D., Zhan G. (2025). Hollow Pt/CeO_2_ nanocatalysts pretreated with pulsed steam for enhanced CO oxidation performance. Mol. Catal..

[53] Song H.C., Han G., Reddy K.P., Choi M., Ryoo R., Park J.Y. (2023). Synergistic interactions between water and the metal/oxide interface in CO oxidation on Pt/CeO_2_ model catalysts. Catal. Today.

[54] Li Y., Liang P., Yu Y., Min X., Wang G., Zhao B., Sun T. (2025). Unravelling the enhanced water-promotion effect for low-temperature CO oxidation over Pt/Ce@SSZ-13 catalyst with highly dispersed Pt-CeO_2_ interfaces. Chem. Eng. J..

[55] El-Bahy Z.M., Alotaibi M.T., El-Bahy S.M. (2022). CO oxidation and 4-nitrophenol reduction over ceria-promoted platinum nanoparticles impregnated with ZSM-5 zeolite. J. Rare Earths.

[56] Yang F., Zhong J., Liu X., Zhu X. (2018). A novel catalytic alkylation process of syngas with benzene over the cerium modified platinum supported on HZSM-5 zeolite. Appl. Energy.

[57] Shilina M.I., Krotova I.N., Udalova O.V., Stolyarov I.P., Cherkashina N.V., Rostovshchikova T.N. (2025). One-step synthesis of PtCo/ZSM-5 catalysts for preferential CO oxidation in a hydrogen excess. Russ. Chem. Bull..

[58] Ivanin I.A., Krotova I.N., Udalova O.V., Zanaveskin K.L., Shilina M.I. (2021). Synergistic catalytic effect of cobalt and cerium in the preferential oxidation of carbon monoxide on modified Co/Ce/ZSM-5 zeolites. Kinet. Catal..

[59] Hadjiivanov K.I., Vayssilov G.N. (2002). Characterization of oxide surfaces and zeolites by carbon monoxide as an IR probe molecule. Adv. Catal..

[60] Shilina M.I., Udalova O.V., Nevskaya S.M. (2013). Synergism in the actions of a transition metal cation and a Lewis acid in the liquid and gas phase catalytic conversion of alkanes over modified ZSM-5 zeolites under mild conditions. Kinet. Catal..

[61] Chakarova K., Mihaylov M., Hadjiivanov K. (2005). FTIR spectroscopic study of CO adsorption on Pt–H–ZSM-5. Microporous Mesoporous Mater..

[62] Chakarova K., Hadjiivanov K., Atanasova G., Tenchev K. (2007). Effect of preparation technique on the properties of platinum in NaY zeolite: A study by FTIR spectroscopy of adsorbed CO. J. Mol. Catal. A Chem..

[63] Aleksandrov H.A., Neyman K.M., Hadjiivanov K.I., Vayssilov G.N. (2016). Can the state of platinum species be unambiguously determined by the stretching frequency of an adsorbed CO probe molecule?. Phys. Chem. Chem. Phys..

[64] DeRita L., Dai S., Lopez-Zepeda K., Pham N., Graham G.W., Pan X., Christopher P. (2017). Catalyst architecture for stable single atom dispersion enables site specific spectroscopic and reactivity measurements of CO adsorbed to Pt atoms, oxidized Pt clusters, and metallic Pt clusters on TiO_2_. J. Am. Chem. Soc..

[65] Shilina M., Udalova O., Krotova I., Ivanin I., Boichenko A. (2020). Oxidation of carbon monoxide on Co/Ce-modified ZSM-5 zeolites: Impact of mixed Oxo-Species. ChemCatChem.

[66] Saveleva V.A., Papaefthimiou V., Daletou M.K., Doh W.H., Ulhaq-Bouillet C., Diebold M., Zafeiratos S., Savinova E.R. (2016). Operando Near Ambient Pressure XPS (NAP-XPS) Study of the Pt Electrochemical Oxidation in H_2_O and H_2_O/O_2_ Ambients. J. Phys. Chem. C.

[67] Kaplin I.Y., Lokteva E.S., Maslakov K.I., Tikhonov A.V., Kharlanov A.N., Fionov A.V., Kamaev A.O., Isaikina O.Y., Maksimov S.V., Golubina E.V. (2022). Ceria-silica mesoporous catalysts for CO preferential oxidation in H_2_-rich stream: The effect of Ce:Si ratio and copper modification. Appl. Surf. Sci..

[68] Zhou X., Zhang H., Sun Y., Gao Z., Chen H. (2023). Defective zeolite TS-1 confined Pt nanoclusters with superior performance for CO and soot catalytic oxidation. Fuel.

