# Supplementary material for: Structure Driven Tuning of the Catalytic Performance of PtCe-Modified Zeolite ZSM-5 in the CO Oxidation
Source: Molecules. 2026 Jan 1;31(1):156. doi: 10.3390/molecules31010156 (PMC12787153; doi:10.3390/molecules31010156)
Supplement: Supplementary file 1 [file molecules-31-00156-s001.zip › molecules-4076404-supplementary.pdf]

Supplementary data

Structure driven tuning of the catalytic performance of PtCe-modified zeolite ZSM-5 in the CO oxidation

Marina Shilina[1],*, Irina Krotova[1], Konstantin Maslakov[1], Stanislava Petrova[1], Olga Udalova[2], Tatiana Rostovshchikova[1]

[1] Lomonosov Moscow State University, 119991 Moscow, Russia; mish@kinet.chem.msu.ru
[2] Semenov Federal Research Center for Chemical Physics, RAS, 119991 Moscow, Russia
* Correspondence: mish@kinet.chem.msu.ru; Tel.: +74959393498, Shilina M.I.

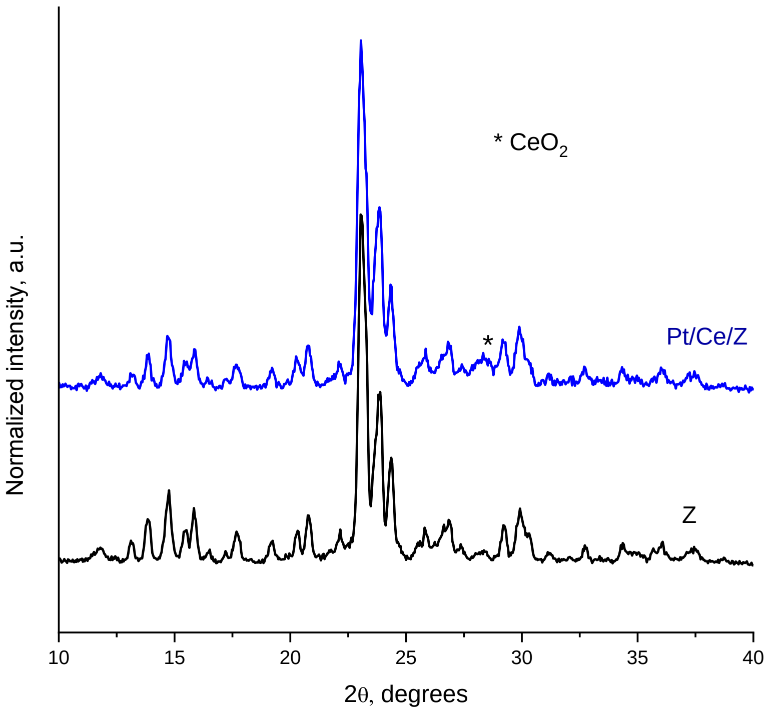

Figure S1. X-ray diffraction patterns of initial ZSM-5 zeolite (Z) and bimetallic Pt/Ce/Z samples. Peaks corresponding to cubic CeO_2 phase ((JCPDS: 43–1002) are denoted with asterisk.

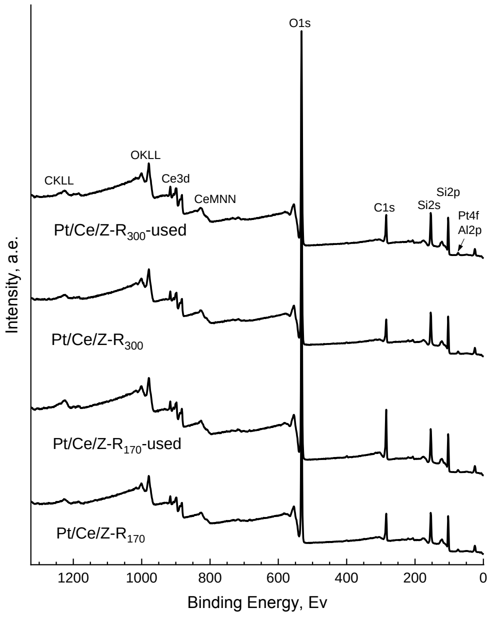

Figure S2. Survey XPS spectra of some Pt/Ce/Z-catalysts
